# Plant Expression of Hydrophobin Fused K39 Antigen for Visceral Leishmaniasis Immunodiagnosis

**DOI:** 10.3389/fpls.2021.674015

**Published:** 2021-05-31

**Authors:** Bruno B. Silva, Eduarda N. F. N. Santos, Lucelina S. Araújo, Arnaldo S. Bezerra, Lívia É. C. Marques, Eridan O. P. Tramontina Florean, Maurício F. van Tilburg, Maria Izabel F. Guedes

**Affiliations:** ^1^Laboratory of Biotechnology and Molecular Biology, Health Sciences Center, State University of Ceará, Fortaleza, Brazil; ^2^Northeast Biotechnology Network (RENORBIO), State University of Ceará, Fortaleza, Brazil; ^3^Department of Biochemistry and Molecular Biology, Federal University of Ceará, Fortaleza, Brazil; ^4^Department of Animal Sciences, Federal Rural University of the Semiarid, Mossoró, Brazil

**Keywords:** visceral leishmaniasis, plant transient expression, recombinant proteins, hydrophobins, neglected tropical diseases, immunodiagnosis

## Abstract

Visceral leishmaniasis is a Neglected Tropical Disease of high mortality caused by the protozoan *Leishmania infantum*. Its transmission cycle is complex, and it has in the domestic dog its main reservoir. The diagnostic tests currently used rely on prokaryotic systems’ proteins, but their low sensitivity increases the disease’s burden. The plant transient expression of recombinant proteins allows the production of complex antigens. However, this system has limited competitiveness against the bacterial production of purified antigens. Thus, we have shown that the *L. infantum* K39 antigen’s fusion to a hydrophobin allows its production for diagnostic tests without the need for intensive purification. The sera of naturally infected dogs specifically detect the semi-purified rK39-HFBI protein. The test validation against a panel of 158 clinical samples demonstrates the platform’s viability, resulting in sensitivity and specificity of 90.7 and 97.5%, respectively. Thus, the use of semi-purified antigens fused to hydrophobins can become the standard platform for large-scale antigens production to expand diagnostic tests for other human and veterinary diseases worldwide.

## Introduction

Visceral leishmaniasis is a Neglected Tropical Disease that annually afflicts 300,000 people worldwide, resulting in the death of 20,000 of them (World Health Organization, 2018; Ruiz-Postigo et al., 2020). In the Americas, visceral leishmaniasis is a zoonotic disease, with the transmission cycle involving the domestic dog (reservoir), the protozoan *Leishmania infantum* (etiological agent) and the sandfly *Lutzomyia longipalpis* (primary vector; Burza et al., 2018). Brazil accounts for 96% of all visceral leishmaniasis cases reported in Latin America (WHO-PAHO, 2018), highlighting the need for new strategies of prophylaxis, diagnosis and treatment for the control of the disease.

The Brazilian government’s main prophylactic measures are the serological surveys followed by the culling of infected dogs (Marcondes and Day, 2019). The epidemiological surveillance is performed in two steps: an immunochromatographic screening test (DPP® Dual Path Platform Biomanguinhos/Fiocruz, Brazil) followed by a confirmatory immunoenzymatic assay (ELISA® kit EIE-LVC Bio-Manguinhos/Fiocruz, Brazil; de Carvalho et al., 2018; Teixeira et al., 2019).

The serological diagnosis of visceral leishmaniasis relies on the recombinant K39 antigen expressed in *Escherichia coli* (Faria and de Andrade, 2012). The K39 protein is predominant in the amastigote stage of *L. infantum*, and it belongs to the kinesin superfamily. These proteins comprise a class of eukaryotic motor proteins involved in microtubule-based movements. The tandem repeats of the kinesins are responsible for protein-protein interaction and its oligomerization inside the cell (Verhey et al., 2011). Tandem repeats proteins from many parasitic protozoans have immunodominant epitopes for antibody production. The number of antibodies that bind to these antigens is proportional to their number of repeats (Goto et al., 2010).

Low-cost diagnostic tests could be developed using less purified heterologous proteins from plants (Buyel, 2019; Marques et al., 2020), an approach that would not be feasible with products from bacterial systems due to the higher antigenicity of the host proteins, demanding their removal by multiple chromatographic steps and increasing the costs for the development of immunodiagnostic tests (Robichon et al., 2011; Tripathi, 2016). The fusion of a plant produced antigen to a hydrophobin partner from *Trichoderma reesei* allows its accumulation at high levels in the plant cell and its subsequent purification by a non-chromatographic and straightforward Aqueous Two-Phase System (ATPS; Linder et al., 2004; Saberianfar and Menassa, 2017).

Thus, the present work describes the plant transient expression of the hydrophobin fused rK39 antigen from *L. infantum*. Upon ATPS, we demonstrated that the construct was partitioned to an rK39-HFBI semi-purified extract. Compared to the purified rK39 produced in *E. coli*, the rK39-HFBI semi-purified extract showed an equivalent performance, which highlights its suitability as a platform for the development of immunodiagnostic tests for canine visceral leishmaniasis.

## Materials and Methods

### Media

YM medium composition was 0.04% Yeast extract, 1.0% Mannitol, 1.7 mM NaCl, 0.8 mM MgSO4, and 2.2 mM K2HPO4. Infiltration media was prepared by the supplementation of YM medium with MES buffer (pH 5.6; Sigma, M3671) and acetosyringone (Sigma, D134406) to a final concentration of 10 mM and 100 μM, respectively. Gamborg’s solution composition was 10 mM MES, 200 μM acetosyringone, 20 g/L sucrose and 3.2 g/L of Gamborg’s B-5 Basal Medium (Sigma, G5893; Kapila et al., 1997).

### Construct Synthesis and Cloning

The *L. infantum* K39 gene (Accession Number: DQ831678.1) was optimized for expression in *Nicotiana benthamiana* and synthesized by BioBasic Inc. (Ontario, Canada). The synthesized construct was flanked by the AttL1 and AttL2 sites to be promptly cloned into the pCAMGate-ER-HFBI expression vector using the Gateway LR Clonase Enzyme Mix II (Invitrogen, 11791020).

This pCAMGate-ER-HFBI plasmid is part of the pCaMGate plant binary expression series. Upstream to its cloning site, the expression cassette encompasses a double enhanced cauliflower mosaic virus 35S promoter, the tCUP translation enhancer, the Pr1b secretory signal peptide from tobacco and the Xpress tag for construct detection. After recombination, the 3' end of the subcloned K39 gene is fused by a 3(GSSS) linker to the HFBI gene. Once expressed, the construct also displays a human c-myc detection/purification tag and a KDEL retention signal at its c-terminus (Pereira et al., 2014).

**Figure 1:**
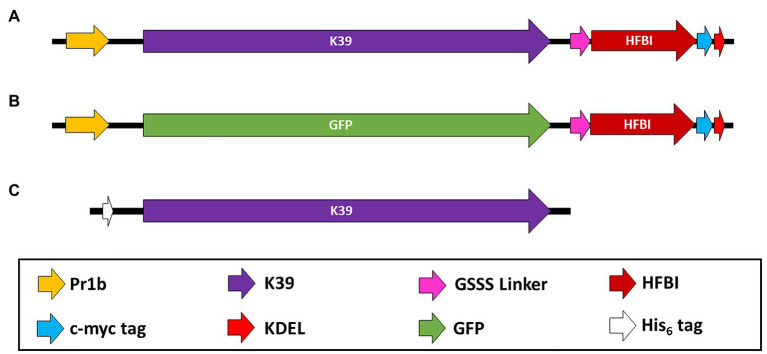
Schematic diagram of the constructs used. **(A)** Construct used for plant expression of the K39 protein from *Leishmania infantum* in the pCAMGate-ER-HFBI vector. **(B)** A construct containing the Green Fluorescent Protein (GFP) as a control for the plant expressed protein in the serological assays. **(C)** Construct used for *Escherichia coli* expression of the K39 protein in the pET-28a vector. Pr1b: tobacco pathogenesis-related protein 1b secretory signal peptide; K39: *L. infantum*’s K39 coding sequence; GSSS: 3(GSSS) linker; HFBI: hydrophobin I gene from the fungus *Trichoderma reesei*; c-myc tag: human c-myc sequence for the detection of the recombinant protein; KDEL: endoplasmatic reticulum retention signal; GFP: Green Fluorescent Protein coding sequence; His_6_-tag: Six histidine residues for recombinant protein detection and purification by immobilized metal affinity chromatography.

The recombination product was transformed into chemically competent *E. coli* DH10B, and a positive clone was selected by colony PCR using vector-specific primers for the 35S promoter (P-35S: CCTTCGCAAGACCCTTCCTCTAT) and nos terminator sequences (T-nos: CCGGCAACAGGATTCAATCTTAA). The pCAMGATE-ER-rK39-HFBI plasmid was transformed into chemically competent *Agrobacterium tumefaciens* LBA4404 (Invitrogen, 18313015) plated into YM medium with the antibiotics kanamycin (50 μg/ml) and streptomycin (100 μg/ml). The same plant expression vector harboring a Green Fluorescent Protein (GFP) was used as a control for the experiments (Figures 1A,B).

### Production of rK39 in *E. coli*

The same K39 coding sequence was cloned into the plasmid pET-28a (Novagen, Darmstadt, Germany) using classical molecular cloning techniques (Figure 1C). The recombinant plasmid obtained was confirmed by capillary sequencing (Helixxa Genomic Services) and then transformed into *E. coli* BL21-DE3 (Invitrogen, C600003).

For the expression of rK39, a pre-inoculum was diluted (1:25) in LB-kanamycin and incubated at 37°C under agitation (250 rpm). When the optical density (OD600 nm) reached 0.6–0.8, the induction process was initiated, using the inducing agent Isopropyl β-D-1-thiogalactopyranoside (IPTG; Sigma, 10724815001) in three different concentrations (0.1; 0.5; and 1 mM). After 3 h of incubation, the bacteria were centrifuged at 8,000 × *g*, 4°C, for 15 min and the resulting pellet resuspended in lysis buffer [200 mM NaCl (Vetec); 20 mM Tris-HCl (USB/Dynamic) pH 8.0; 200 μg lysozyme; 2 mM phenylmethanesulfonyl fluoride (PMSF; Sigma, P7626) with 1% Triton X-100 (USB)]. The samples were sonicated (12 cycles of 15 s) and then centrifuged (8,000 × *g*, 4°C, 15 min). The rK39 protein was purified by immobilized metal affinity chromatography from the supernatant of the bacterial extract. The samples were loaded into a Ni-NTA column (GE Healthcare, United States) previously equilibrated (20 mM Tris pH 8.0; 500 mM NaCl; 10% glycerol; 20 mM imidazole). After the washing step, the retained recombinant proteins were retrieved using an elution buffer (20 mM Tris pH 8.0; 500 mM NaCl; 10% glycerol; 500 mM imidazole; Rodrigues et al., 2005). The purified proteins were analyzed by SDS-polyacrylamide gel electrophoresis (SDS-PAGE) and Western Blotting, using the monoclonal anti-His6 antibody (GE Healthcare Life Sciences) diluted 1:6,000.

### Mice Immunization for the Production of rK39 Antisera

Six female mice (BALB/c), aged 6–8 weeks and weighing 25–30 g, were obtained from the Nucleus of Experimental Biology (Nubex) of the University of Fortaleza (Unifor). The animals were kept under controlled temperature and humidity conditions, with a light-dark cycle of 12 h. Throughout the experiment, feed and water were provided ad libitum.

After acclimatization, the animals were subcutaneously immunized with 20 μg of the purified rK39 protein associated with Freund’s complete adjuvant (Sigma-Aldrich), receiving boosters with Freund’s incomplete adjuvant on the 21st and 35th days after the beginning of the immunization. To obtain the antisera, the animals had their blood collected by the retro-orbital plexus on day 0 (pre-immune) and the 42nd day after the start of immunization (Mello et al., 2011). The sera were stored at −20°C until its use.

### *Nicotiana benthamiana* Agroinfiltration

A single *A. tumefaciens* colony harboring the plasmid pCAMGATE-ER-rK39-HFBI or pCAMGATE-ER-GFP-HFBI was picked from fresh plates to prepare each inoculum. The same was done to an *A. tumefaciens* clone harboring the coding sequence to the p19 silencing inhibitor from Cymbidium ringspot virus (CymRSV). After overnight growth, both inocula were diluted (1:1,000) into infiltration media, and they were then incubated at 28°C (220 rpm) overnight. After reaching an OD600 of 0.8–1.0, the cultures were centrifuged (4,000 × *g*; 30 min; 22°C), and the resulting pellets were resuspended into MMA solution to an OD600 of 1.0. After 1-h incubation under gentle shaking at room temperature, the resuspended cells were mixed (1:1) for plant infiltration. The leaves of 6–8 weeks old *N. benthamiana* plants were infiltrated using a needleless syringe. Control plants were infiltrated only with the p19 culture (Pereira et al., 2014). The plants were kept at 25°C with a photoperiod of 16 h and on hydroponic solution along with the whole experiment.

### rK39-HFBI Extraction and ATPS Purification

The infiltrated leaves were collected at 2, 4, 6, and 8days post infiltration (d.p.i.). On these days, leaves of three different plants were collected. A 4 cm^2^ piece of each leaf was weighted and then grounded on a microtube with three volumes of ice-cold extraction buffer [PBS with 0,1% Triton™ X-100 (Sigma, T8787) and 10 mM PMSF (Sigma, P7626)]. The macerated leaves were centrifuged (10,000 × *g*; 10 min; 4°C), and the supernatant was stored at −20°C for posterior analysis. All the experiments were performed on three independent batches of plants.

For ATPS purification, the agroinfiltrated leaves were harvested, weighted, and grounded on a mortar with liquid nitrogen. Six volumes of buffer were added to the powdered leaves that were then incubated for 1 h at 4°C under gentle shaking. The leaves were filtered on mousseline cloth and afterward centrifuged (6,000 × *g*; 30 min; 4°C). The supernatant was collected on new tubes, to which 2, 4, or 8% (v/v) of Triton™ X-114 (Sigma, X114) was added and homogenized by vortexing. The tubes were incubated in a heat block at 30°C until phase separation was stable (approximately 30 min or until the Triton X-114 lower phase showed a 10-fold increase from the initial surfactant volume). The upper aqueous phases were stored for posterior analysis, and one volume of isobutanol (Sigma, 33064) was added to each tube. After homogenization and centrifugation (6,000 × g; 15 min; 22°C), the upper isobutanol phase and the insoluble middle-phase were discarded. The aqueous lower phase (rK39-HFBI semi-purified extract) was submitted for analysis (Joensuu et al., 2010; Reuter et al., 2014, 2016).

### Protein Quantification, SDS-PAGE, and Western Blotting

Total soluble proteins (TSP) of all extracts were quantified (Bradford, 1976) and analyzed using a 12% SDS-PAGE (Laemmli, 1970). For the protein expression kinetics, 20 μg of leaf extract of each time point were submitted to Western Blotting analysis using mouse antisera against *E. coli* expressed His-tagged rK39 (1:250 dilution) and the peroxidase-conjugated anti-Mouse IgG (1:5,000) (Invitrogen, G21040).

The same antisera were used to discard cross-reactivity against *N. benthamiana* and *A. tumefaciens* proteins. For this, 10 μg of TSP from rK39-HFBI infiltrated plants were analyzed and compared to the same amount of TSP from control plants: p19-only infiltrated leaves and GFP-HFBI infiltrated leaves. Around 500 ng of the His-tagged rK39 protein purified from *E. coli* were used as a positive control.

For western blottings using the sera of dogs naturally infected by *L. infantum*, the pooled sera were diluted (1:250) and incubated with the nitrocellulose membrane for 1 h at 37°C. Peroxidase conjugated anti-Dog IgG (Sigma, SAB3700101) was used as the second antibody (1:5,000), and Clarity™ Western ECL Substrate (Bio-Rad, 1705060) was used for membrane development. For c-myc detection, a monoclonal anti-human c-myc antibody (Sigma, M4439) was used as the primary antibody (1:2,500), and the membrane was developed using 3,3'-Diaminobenzidine (Sigma, D8001) solution.

### rK39-HFBI PTA-ELISA for Canine Visceral Leishmaniasis Diagnosis

The population of this study comprised the sera from 118 visceral leishmaniasis positive dogs (DPP positive followed by EIE-LVC positive tests), 20 false-positive sera (DPP positive followed by EIE-LVC negative tests), and 20 negative sera (DPP negative), according to the protocol adopted by the Brazilian government for the diagnosis of canine visceral leishmaniasis (de Carvalho et al., 2018).

The rK39-HFBI semi-purified extract was diluted into coating buffer (0.1 M sodium carbonate, pH 9,5) to the concentration of 1 μg/ml. Then, 100 μl of the diluted extract was used to coat each microplate well (Sigma, M9410). After overnight coating, the plates were washed with PBS-T [0.05% Tween® 20 (Sigma, P9416)], blocked for 1 h with 1% gelatin (Sigma, G6650) and then washed three times with PBS-T. The canine sera were individually diluted in PBS (1:160) and tested in duplicates. After incubating the sera for 1 h at 37°C, another washing step was performed, and 100 μl of peroxidase-conjugated anti-Dog IgG (Sigma, SAB3700101; 1:5,000) was added. After a final washing step, 100 μl of TMB solution (Thermo, 34028) were added to each well, and the plates were incubated for 20 min in the dark. The reaction was stopped with 100 μl of 2 M H_2_SO_4_ solution and the absorbance at 450 nm was read using a microplate reader (Synergy™ 2, Biotek; Florindo et al., 2002). The same procedures were followed for the analysis of the rK39 purified protein.

### Statistical Analysis

The ELISA’s cut-off values and the sensitivity and the specificity of each recombinant antigen were estimated using the Receiver Operating Characteristic (ROC) curves generated by MedCalc® statistical software 17.2. The pairwise comparison of ROC curves was done according to DeLong et al. (1988). Statistical analysis was performed by one-way (ANOVA) using the Kruskal-Wallis multiple comparison test. All the statistical analyses and graphs were generated by the GraphPad Prism 7 release 6.0.

### Ethical Statements

All the experiments were conducted following the guidelines of the State University of Ceará Institutional Animal Care and Use Committee (protocols 3630450/2015 and 7255099/2017).

## Results

### rK39-HFBI Protein Analysis in Agroinfiltrated *N. benthamiana*’s Leaves

To produce the rK39 antigen fused to a hydrophobin, we infiltrated the leaves of 6–8 weeks old *N. benthamiana* with an *A. tumefaciens* LBA4404 culture transformed with a binary plasmid containing the construct rK39-HFBI.

**Figure 2:**
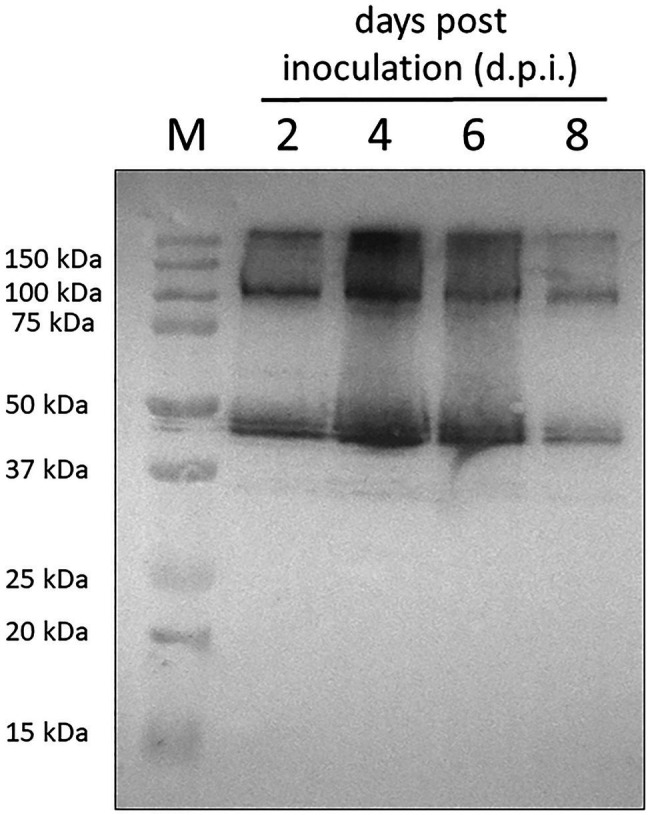
Evaluation of rK39-HFBI expression in infiltrated leaves. Western blotting against 20 μg of total soluble proteins (TSPs) from rK39-HFBI infiltrated leaves at 2-, 4-, 6-, and 8-days post infiltration (d.p.i.).

After infiltration, the best time for protein accumulation was empirically determined. For rK39-HFBI, the protein expression dynamics’ evaluation required normalization by the leaf area instead of the fresh plant weight, since necrosis of the leaf tissue was initiated at 6 d.p.i. and progressed until the material was infeasible for analysis at 10 d.p.i. (data not shown). The necrotic process was compatible with the lower level of expression detected at 6 and 8 d.p.i., even when equal amounts of total soluble protein were loaded into each lane for western blotting analysis. The Western blotting analysis of the leaves’ TSP evidenced a band of molecular weight close to 50 kDa, compatible with the 48.1 kDa expected size for the K39 protein when expressed in the pCamGATE-ER-HFBI vector (Figure 2).

**Figure 3:**
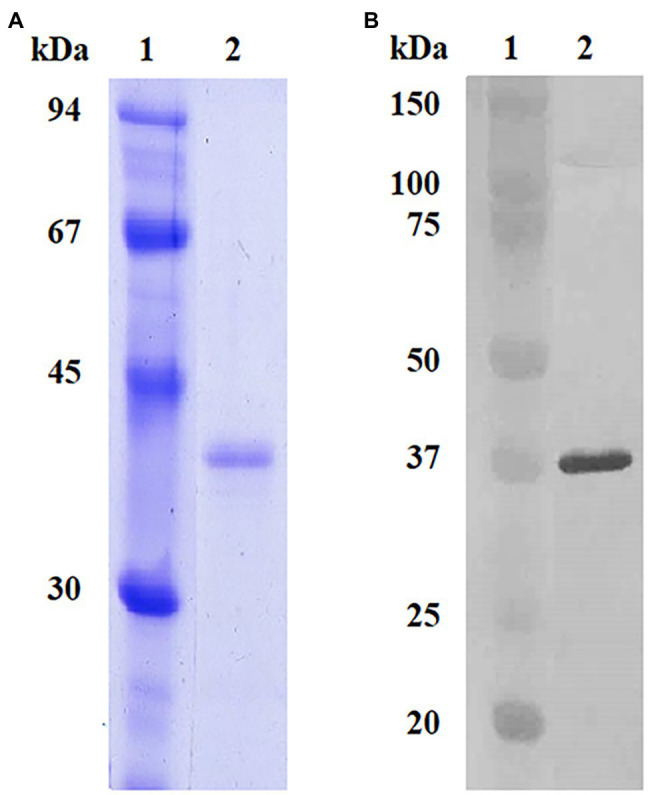
SDS-PAGE and Western blotting of the recombinant proteins produced in *E. coli* and *Nicotiana benthamiana*. **(A)** Coomassie Brilliant Blue G-250 stained SDS-PAGE from the purified rK39 produced in *E. coli*. **(B)** Western blotting of the purified rK39 produced in *E. coli* using an anti-His antibody.

As a control for our assays and comparison of test performance, the rK39 protein was also produced and purified from *E. coli* culture. The use of a monoclonal antibody specific for the His_6_ tag of the rK39 antigen produced in *E. coli* enabled the detection of a single band of 37 kDa, equivalent to the size expected for the K39 protein of *L. infantum* when expressed in the pET-28a vector (Figure 3).

### Western Blotting Analysis of Total Soluble Proteins From rK39-HFBI Infiltrated Leaves Against the Sera From rK39 Immunized BALB/c Mice and Naturally Infected Dogs

To assess whether host plant proteins would affect the specific recognition of the rK39-HFBI antigen, we did a Western blotting of the total soluble proteins from infiltrated and control leaves using BALB/c mice’s sera raised against the rK39 antigen produced in *E. coli*, as well as sera samples from dogs diagnosed with visceral leishmaniasis.

**Figure 4:**
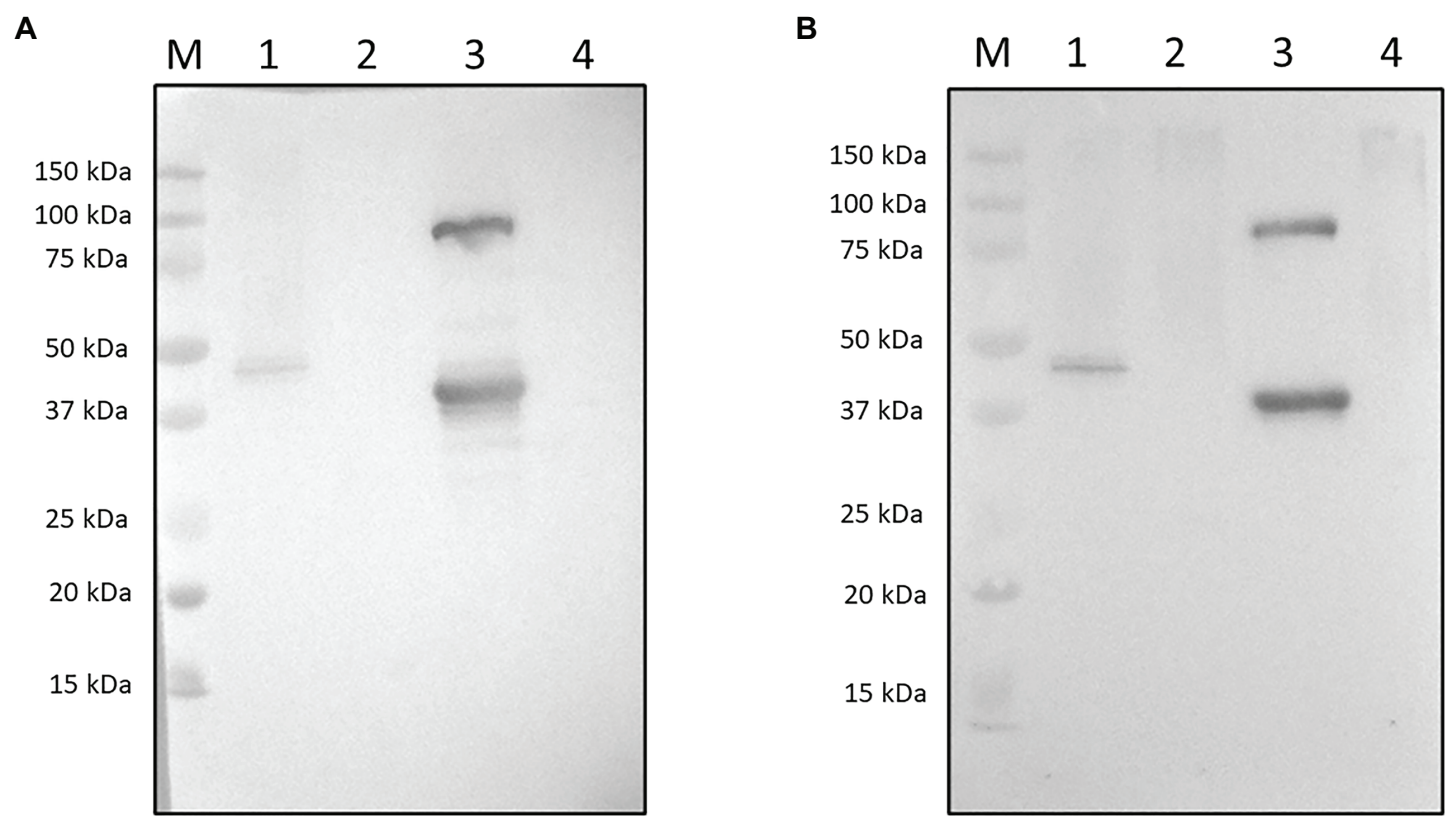
Western Blotting of TSPs from rK39-HFBI infiltrated leaves. **(A)** Using mice antisera (1:250 dilution): (1) 10 μg of total soluble proteins from rK39-HFBI infiltrated leaves; (2) About 10 μg of total soluble proteins from p19-only infiltrated control plants; (3) Around 500 ng of His-tagged purified rK39; and (4) About 10 μg of total soluble proteins from GFP-HFBI infiltrated control plants. **(B)** Against sera of *L. infantum* naturally infected dogs (1:250 dilution): (1) 10 μg of total soluble proteins from rK39-HFBI infiltrated leaves; (2) Around 10 μg of total soluble proteins from p19-only infiltrated control plants; (3) Around 500 ng of His-tagged purified rK39; and (4) About 10 μg of total soluble proteins from GFP-HFBI infiltrated control plants.

Western blotting with control plants was done to discard any antibody cross-reactivity against the host plant or the *A. tumefaciens* proteins (Figure 4A). As it can be seen, only bands from the rK39-HFBI plant extract and the prokaryotic K39 control were specifically detected. No reaction could be seen against equivalent amounts of control plant extracts (p19 infiltrated) or with plants agroinfiltrated with a hydrophobin-fused control protein. Similar results were observed using the antisera from dogs naturally infected with *L. infantum* (Figure 4B).

### Aqueous Two-Phase System Purification of the rK39-HFBI Protein

**Figure 5:**
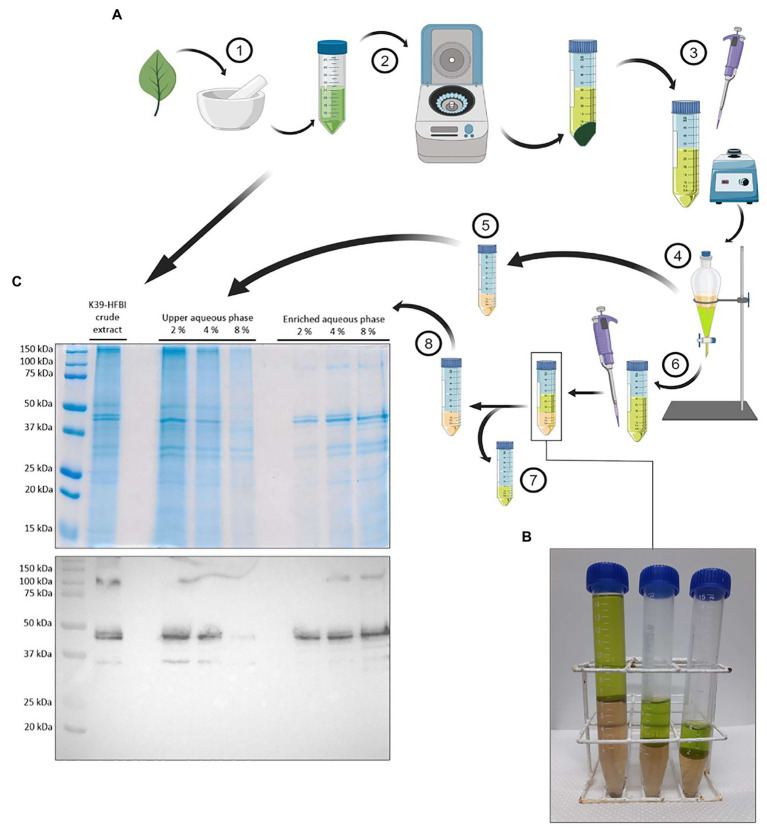
Aqueous Two-Phase System (ATPS) separation for rK39-HFBI. **(A)** ATPS process: (1) rK39-HFBI infiltrated leaves are ground on liquid nitrogen and resuspended in 6 volumes of buffer; (2) rK39-HFBI crude extract is centrifuged for removal of cell debris; (3) The surfactant Triton X-14 is added (2, 4, or 8%, v/v) to the supernatant and homogenized; (4) This mixture is incubated at 30°C for phase-separation; (5) The aqueous upper phase is reserved for posterior analysis; (6) Isobutanol is added to the lower surfactant phase, homogenized and centrifuged for a new phase separation; (7) The isobutanol upper phase is discarded; and (8) The lower aqueous rK39-HFBI semi-purified fraction is analyzed. **(B)** The final volume of rK39-HFBI semi-purified fraction (lower phase) is proportional to the amount of Triton X-114 used (8, 4, and 2% from left to right). **(C)** SDS-PAGE and Western Blotting of the ATPS resulting fractions. K39-HFBI crude extract: 10 μg; ATPS fractions: volume loaded proportional to the final volume of each fraction (from left to right: upper aqueous phase (20, 10, and 5 μl), semi-purified fraction: (5, 10, and 20 μl). Created with Biorender.

The addition of the surfactant Triton X-114™ to the plant crude extract allows the partitioning of the HFBI fused protein away from host proteins, yielding an upper aqueous phase and an rK39-HFBI semi-purified extract (Figure 5A). In this study, the use of three concentrations of surfactant (2, 4, and 8%) resulted in different yields of rK39-HFBI in the semi-purified extracts. It is noteworthy that the rK39-HFBI semi-purified extract’s final volume depends on the amount of surfactant used (Figure 5B). After phase separation, the Triton X-114 phase displays a 10-times increase in its initial volume. Thus, each of these phases’ final volume normalized the SDS-PAGE and western blotting shown (Figures 5C).

**Figure 6:**
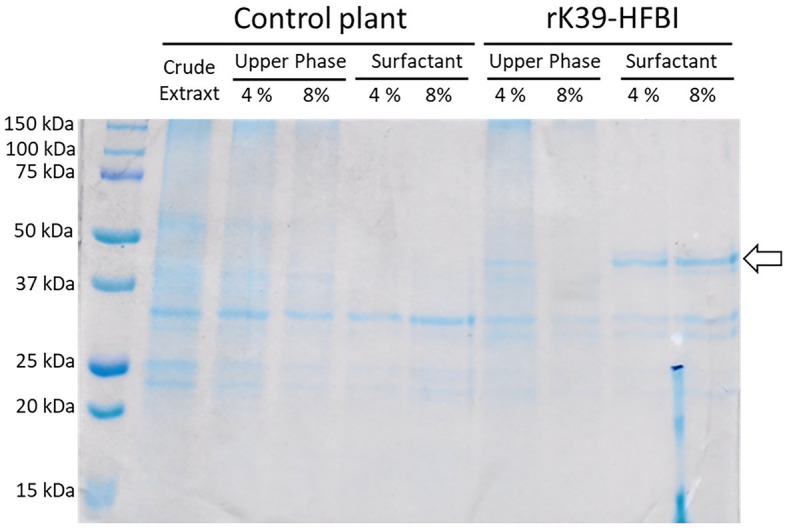
SDS-PAGE of the ATPS resulting fractions from control plants and from rK39-HFBI infiltrated leaves. Control plant crude extract: 10 μg. ATPS fractions: volume loaded proportional to the final volume of each fraction [upper aqueous phase: 4% (20 μl) and 8% (10 μl), semi-purified fraction: 4% (10 μl) and 8% (20 μl)]. The arrow indicates the K39-HFBI construct (~48 kDa), absent in the control plants.

As can be seen, at 2% of surfactant, a significant amount of the rK39-HFBI construct is lost at this upper aqueous phase; however, an increased surfactant concentration displaces the rK39-HFBI construct of this fraction toward the surfactant phase, as seen at 4 and 8% of Triton X-114 (Figure 5C). We chose to work with the intermediary concentration of 4%, as it had a good balance between the final volume of rK39-HFBI semi-purified fraction and the amount of surfactant required. When the p19 control plants and the rK39-HFBI infiltrated leaves are submitted to ATPS, only in the latter, it is possible to visualize a specific band at 48 kDa (Figure 6), demonstrating the successful partitioning of the construct to the surfactant phase.

### Evaluation of rK39-HFBI Semi-Purified Fraction for Canine Visceral Leishmaniasis Diagnosis by Plate Trapped Antigen-ELISA

To validate the rK39-HFBI construct for *L. infantum* infection diagnosis, we tested it against a panel of positive and negative sera for canine visceral leishmaniasis. The same samples were also tested against the rK39 produced in *E. coli* to compare the performance of both platforms, prokaryotic and eukaryotic, in the diagnosis of visceral leishmaniasis. The diagnostic performance of each recombinant antigen is summarized.

**Figure 7:**
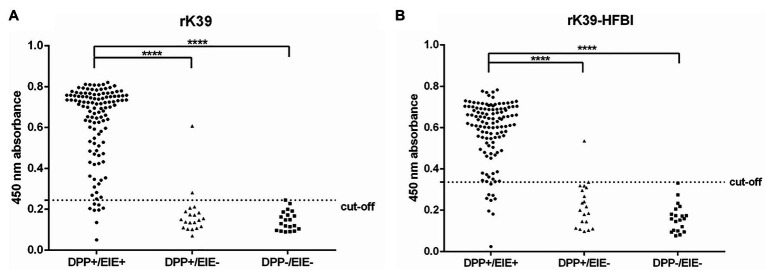
ELISA with canine positive and negative sera for *L. infantum* infection according to the Brazilian government protocol. **(A)** Samples evaluated using plates coated with 100 ng/well of rK39 purified protein. **(B)** Samples evaluated using plates coated with 100 ng/well of rK39-HFBI semi-purified extract. DPP+: Dual Path Platform positive for canine visceral leishmaniasis; DPP-: Dual Path Platform negative for canine visceral leishmaniasis; EIE+, Immunoenzymatic assay positive for canine visceral leishmaniasis; EIE-, Immunoenzymatic assay negative for canine visceral leishmaniasis. ANOVA followed by Kruskal-Wallis test, **** < 0.0001 [(DPP-) vs. (DPP + /EIE+); (DPP + /EIE-) vs. (DPP + /EIE+)].

Eleven samples from the DPP+/EIE+ group differed in our analyzes. Of these, two samples were negative only for rK39 (Figure 7A) and three samples for rK39-HFBI (Figure 7B). The other six samples were negative for both recombinant proteins. When the results from the rK39-HFBI and rK39 ELISA tests were compared for the DPP+/EIE‐ group (considered healthy animals by the Brazilian government protocol), we verified that one sample was positive in both of our tests, while another was positive only for the rK39.

**Figure 8:**
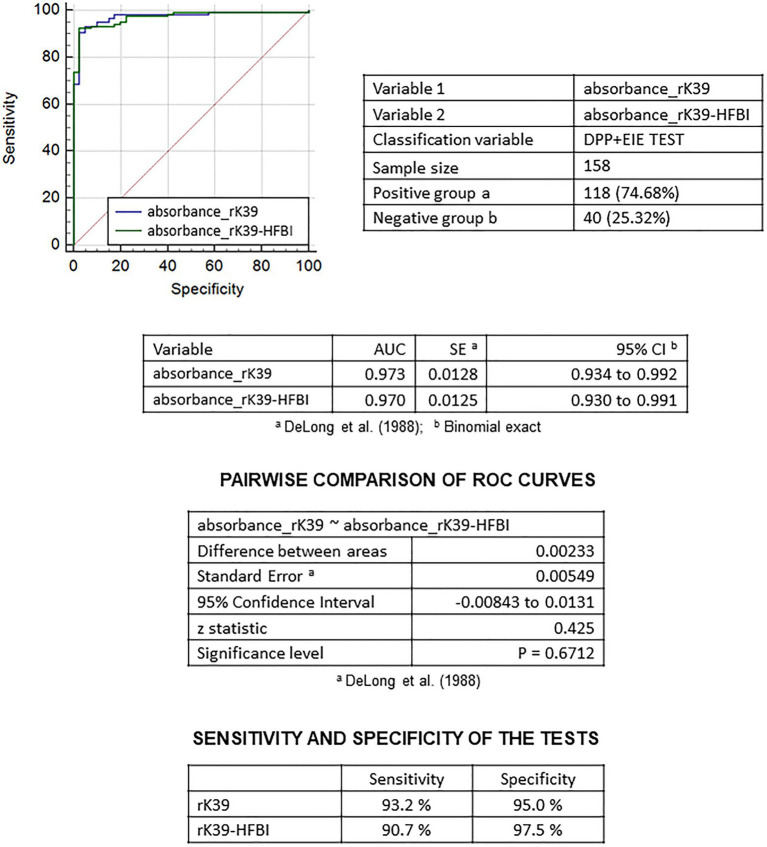
Sensitivity, specificity, and pairwise comparison of Receiver Operating Characteristic (ROC) curves of rK39 and rK39-HFBI tests. The calculations considered positive and negative for visceral leishmaniasis samples from a sera panel tested according to the serological diagnostic protocol adopted by the Ministry of Health in Brazil.

Although the ROC curve analysis of the area under the curve (AUC) showed similar performance of rK39-HFBI (AUC = 0.970) compared to rK39 (AUC = 0.973), the data showed higher specificity for the rK39-HFBI semi-purified extract (97.5%) rather than the purified prokaryotic antigen (95.0%). Our results have shown, however, that the semi-purified rK39-HFBI test had a lower sensitivity (90.7%) compared to its prokaryotic counterpart (93.2%; Figure 8).

## Discussion

In this work, the K39 antigen from *L. infantum* was transiently expressed in plants. After subcloning the K39 gene into the expression vector, each newly produced batch took only 6 days from *A. tumefaciens* growth to the purification and coating of microplates with the antigen for visceral leishmaniasis diagnosis. The use of ATPS to reduce purification costs resulted in the development of a semi-purified rK39-HFBI test, which had a performance equivalent to the purified protein produced in *E. coli*. Thus, it highlights the transient expression system’s potential for the fast and straightforward production of antigens for diagnostic tests against visceral leishmaniasis, as well as other human and veterinary diseases.

The high expression of rK39-HFBI at 4 d.p.i. falls into the time range reported in the literature for the most significant accumulation of transiently expressed proteins (4–10 d.p.i.; Shamloul et al., 2014). The expression of this protein was accompanied by necrosis of the leaves starting at 6 days post infiltration. The hypersensitive response of the plant cells against the *A. tumefaciens* infection could be the primary mechanism responsible for the necrotic process (Leuzinger et al., 2013; Cana-Quijada et al., 2018). These results are not unusual since different kinds of recombinant proteins might exert toxic effects over the leaf cells when overexpressed. In a murine model of malaria, Webster et al. (2009) reported maximum yields of the recombinant *Plasmodium yoelii* merozoite surface protein 4/5 (PyMSP4/5) at 2–7 d.p.i. accompanied by necrotic spots in the infiltrated leaves of *N. benthamiana*, corroborating our findings. Diaz et al. (2020) also report progressive damage to the *N. benthamiana*’s leaf tissue due to the *A. tumefaciens* mediated transient expression of a saposin-like protein from *Trichomonas vaginalis* (rTvSaplip12). These authors report that a lower concentration of agrobacteria reduced the severity of the symptoms in infiltrated leaves but also led to a lower expression of rTvSaplip12.

The findings that the sera from K39 immunized mice, or naturally infected dogs, only reacted with the rK39-HFBI crude extract, reinforce that host protein contaminating a plant expressed antigen extract would not interfere with its use for immunodiagnosis. As control plant extracts showed no reaction to infected dogs’ antisera, it reassured the potential of transient plant expression as a reliable system for the production of antigens for immunodiagnosis without the need for intensive purification.

Plate Trapped Antigen-ELISA (PTA-ELISA) tests from plant extracts have already been demonstrated as a stable platform for low-cost detection of virus (Mowat and Dawson, 1987; Nascimento et al., 2017). Thus, it could be used to expand access to visceral leishmaniasis diagnosis in low-income countries. Once the recombinant protein was transiently expressed, the plant extract could be easily used for coating microplates for serological diagnostic.

A significant concern toward PTA-ELISA is the stability of this type of test based on non-purified antigens. However, stability of up to 20 months at room temperature has been described for a PTA-ELISA for the detection of plant viruses (Nascimento et al., 2017). Another disadvantage of using total soluble proteins in a PTA-ELISA is that it can lead to a lower sensitivity as the recombinant protein is diluted into the host proteins pool (Garnsey and Cambra, 1993), and the plant compounds could interfere with the reaction.

Therefore, to attain a simple way to concentrate the *L. infantum* K39 protein from the plant crude extract, we fused this protein to the hydrophobin I gene from the fungus *Trichoderma reesei*. The hydrophobins are a class of proteins with tensioactive properties. Once exposed to hydrophobic-hydrophilic interfaces, the hydrophobin monomers undergo conformational changes and associate to form an amphipathic layer (Bayry et al., 2012; Berger and Sallada, 2019). By adding different amounts of the surfactant Triton X-114™ to the plant crude extract, we could concentrate the rK39-HFBI construct. This process, termed ATPS (Linder et al., 2004), allows the partitioning of the HFBI fused protein away from host proteins, yielding an upper aqueous phase and an rK39-HFBI semi-purified extract.

Plant expression systems are particularly advantageous for the production of hydrophobin fused proteins. These fusion partners induce the accumulation of the heterologous protein expressed, increasing their yield and stability in protein bodies. Besides, the presence of few plant proteins with similar affinity to the surfactant results in a higher purity of the hydrophobin semi-purified extract when produced in a plant-based platform (Reuter et al., 2016).

Joensuu et al. (2010) were the first to report the biotechnological potential of hydrophobin fusions for the production and purification of recombinant proteins transiently expressed in plants. In their work, the fusion of HFBI to the c-terminus of GFP doubled its production over the unfused control (approximately 38 and 18% of total soluble proteins, respectively). Despite the necrotic process seen in our work, these authors report that the HFBI fusion was able to prevent the necrosis of the infiltrated *N. benthamiana* leaves and enabled the purification of GFP-HFBI at 91% purity, yielding up to 10 mg/ml of purified recombinant protein.

The rK39-HFBI semi-purified fraction allowed the development of a Plate Trapped Antigen-ELISA for canine visceral leishmaniasis diagnosis. However, since our data relies on a serological panel, its validation against a gold standard (parasitological test) or a 100% specific test (qPCR; Nunes et al., 2015; de Carvalho et al., 2018) still is required. Despite this, our test has shown a good concordance with the results from the official protocol adopted by the Brazilian government, and it shows that plant-produced rK39-HFBI could be used for the diagnosis of visceral leishmaniasis.

An increasing number of Leishmania recombinant antigens have been developed and evaluated for serodiagnosis (Farahmand and Nahrevanian, 2016; Salles et al., 2017; Kühne et al., 2019). The low sensitivity of the tests currently used is the major drawback for the serological diagnosis of visceral leishmaniasis (Teixeira et al., 2019). However, diagnostic tests based on recombinant proteins allow the use of multiple proteins (isolated or as chimeric proteins) as a strategy for improving the performance of the produced test (Travi et al., 2018).

The expression of antigens in plants is a tool mainly used to produce antigens for vaccine development. In this context, the successful production of antigens from protozoa such as *Trypanosoma cruzi*, *Plasmodium falciparum*, and *Toxoplasma gondii* (the etiological agents of Chagas disease, malaria, and toxoplasmosis, respectively), have been described (Mamedov et al., 2019; Ramos-Vega et al., 2021). This platform’s potential for the production of diagnostic antigens is still poorly explored (Schillberg et al., 2019), which led us to assess its features. This paper is the first work, to our knowledge, that used the plant transient expression system to produce a protozoan antigen to develop a diagnostic test.

The plant transient expression platform has shown great potential for producing and assessing new antigens for Neglected Tropical Diseases diagnosis even at a bench-scale. Since multiple proteins could be simultaneously produced (Chen et al., 2014) and tested against a panel of patient sera, this platform facilitates the discovery and validation of new biomarkers for disease diagnosis at low-resource research settings. Furthermore, the fusion of a hydrophobin partner to the produced antigen favors its immobilization into Point-of-Care devices (Zhao et al., 2007; Piscitelli et al., 2017; Zhang et al., 2018), for the development of diagnostic devices technically feasible, affordable, and adequate to different clinical settings (Karl et al., 2015), which also eliminates the need to remove the purification tag by additional steps (Islam et al., 2019).

This work aimed to evaluate the semi-purified rK39-HFBI diagnostic performance for the serological diagnosis of canine visceral leishmaniasis. The promising results found with the K39 antigen of *L. infantum* have opened the way for (ongoing) tests with other protozoan antigens and antigens from viral diseases like dengue and zika fever, and even COVID-19. Our group is completing a detailed cost-benefit analysis of this platform and also evaluating the test’s stability over 1 year to establish its shelf life.

A detailed analysis of the production costs was not within the scope of this work, but our data reinforce that plant transient expression of antigens can be a competitive platform for developing diagnostic tests based on semi purified antigens. Based on our experiments’ usual yields, we estimate that from 16 g of leaf tissue (approximately 4–5 *N. benthamiana* plants), we could obtain enough semi purified rK39-HFBI protein to coat 200 units of 96-well microplates and test at least 9,000 dogs. This level of performance can be further enhanced by optimizing the production process of the recombinant antigen (Grosse-Holz et al., 2018; Peyret et al., 2019; Sainsbury, 2020).

Thus, we have demonstrated that the transient expression of the hydrophobin-fused K39 antigen allows its use to diagnose canine visceral leishmaniasis. The fusion to the HFBI partner allows the partial purification of the antigen at low-cost, and the contaminant host proteins present on this extract do not interfere with the specific diagnosis of the disease. The high yields at bench-scale also support the use of this platform to produce and validate new antigens for the diagnosis of other human and veterinary diseases.

## Data Availability Statement

The raw data supporting the conclusions of this article will be made available by the authors, without undue reservation.

## Ethics Statement

The animal study was reviewed and approved by Ethics Committee on Animal Use, State University of Ceará, Fortaleza, Brazil.

## Author Contributions

BS, ES, ET, MT, and MG designed the experiments. BS and ES performed the experiments and wrote the manuscript. LA performed the mice immunizations and helped in the serology tests. AB and LM helped in the production and purification of the proteins. BS, ES, LA, AB, LM, ET, MT, and MG revised and edited the manuscript. All authors contributed to the article and approved the submitted version.

### Conflict of Interest

The authors declare that the research was conducted in the absence of any commercial or financial relationships that could be construed as a potential conflict of interest.
